# The Effect of Ginger and Its Sub-Components on Pain

**DOI:** 10.3390/plants11172296

**Published:** 2022-09-02

**Authors:** Suyong Kim, Chunhoo Cheon, Bonglee Kim, Woojin Kim

**Affiliations:** 1 Department of Physiology, College of Korean Medicine, Kyung Hee University, Seoul 02447, Korea; 2 Cancer Preventive Material Development Research Center, College of Korean Medicine, Kyung Hee University, Seoul 02447, Korea; 3 Korean Medicine-Based Drug Repositioning Cancer Research Center, College of Korean Medicine, Kyung Hee University, Seoul 02447, Korea

**Keywords:** [6]-gingerol, [6]-shogaol, ginger, pain, *Zingiber officinale* Roscoe

## Abstract

*Zingiber officinale* Roscoe (ginger) has long been used as an herbal medicine to treat various diseases, and its main sub-components, [6]-gingerol and [6]-shogaol, were also reported to have anti-inflammatory, anti-oxidant, and anti-tumor effects. However, their effects on various types of pain and their underlying mechanisms of action have not been clearly analyzed and understood yet. Thus, in this review, by analyzing 16 studies that used *Z. officinale*, [6]-gingerol, and [6]-shogaol on mechanical, spontaneous and thermal pain, their effects and mechanisms of action have been analyzed. Pain was induced by either nerve injury or chemical injections in rodents. Nine studies analyzed the analgesic effect of *Z. officinale*, and four and three studies focused on [6]-gingerol and [6]-shogaol, respectively. Seven papers have demonstrated the underlying mechanism of action of their analgesic effects. Studies have focused on the spinal cord and one on the dorsal root ganglion (DRG) neurons. Involvement and change in the function of serotonergic receptors (5-HT_1A_, _B_, _D_, and _5A_), transient receptor potential vanilloid 1 (TRPV1), N-methyl-D-aspartate (NMDA) receptors, phosphorylated extracellular signal-regulated kinase 1/2 (pERK1/2), histone deacetylase 1 (HDAC1), voltage-gated sodium channel 1.8 (Na_v_1.8), substance P (SP), and sciatic nerve’s morphology have been observed.

## 1. Introduction

*Zingiber officinale* Roscoe is a perennial herb from a member of the Zingiberaceae family [1], and it is known to be rich in various chemical constituents, such as phenolic compounds, terpenes, polysaccharides, lipids, organic acids, and raw fibers [2]. Among the main phenolic compound, gingerols, which are a mixture containing the 3-methoxy-4-hydroxyphenyl functional group, induce *Z. officinale*’s spicy taste and are present in 85 types [3]. Gingerols can be divided into gingerols, shogaols, paradols and zingerones. Among them, gingerols and shogaols are known as the most important physiological active ingredients for *Z. officinale*, of which [6]-gingerol and [6]-shogaol are the main compounds [4].

In the international association for the study of pain (IASP), pain is defined as “an unpleasant sensory and emotional experience associated with, or resembling that associated with, actual or potential tissue damage.” Additionally, pain extends its meaning to personal experiences affected by biological, psychological, and social factors [5]. Pain is present in various forms, such as acute and chronic [6], neuropathic [7], inflammatory [8], and cancer [9] pain. To manage these various types of pain, diverse analgesics are used. Among them, opioids and nonsteroidal anti-inflammatory drugs (NSAIDs) are one of the most widely used pain-reducing drugs in the world. In 2012, 6.8% of the 4.2 billion prescriptions prepared in the United States were opioids [10], and from 2001 to 2009, the number of people who prescribed NSAIDs more than doubled [11]; however, both opioids and NSAIDs have side effects such as hormone imbalance [12], tolerance and dependence [13], nausea, dyspepsia and gastrointestinal ulceration [14]. Thus, efforts to find an optimal analgesic drug that has no or fewer side effects than the currently used analgesics are still needed.

*Z. officinale,* ginger, has long been widely used as an herbal medicine for the prevention and treatment of various diseases [15,16,17], as it has also been reported to show no toxic effects [18]. In clinical studies, it has been reported to alleviate diseases such as diabetes [19,20,21], obesity [22], cancer [23], nausea and vomiting [24]. Furthermore, although low in numbers, *Z. officinale* has also been demonstrated to be effective against different types of pain in humans. Its administration with NSAIDs have decreased migraine attack compared to the placebo-treated group [25]. A systematic review has reported the efficacy of *Z. officinale* to treat primary dysmenorrhea [26], and a clinical report has demonstrated that osteoarthritis patients receiving both *Z. officinale* extract and ibuprofen showed significantly reduced pain [27]. Although more than ten papers, which have focused on the effect of *Z.* *officinale* and its sub-components on pain have been published, to date no study has summarized the effect of *Z. officinale* and its sub-components on various types of pain.

From the past, our lab has focused our efforts to understand the pathophysiological and curative mechanism of different types of pain, such as chemotherapy-induced neuropathy (CIPN) [28,29] and diabetic-induced neuropathic pain [30]. In our previous study, the water extract of *Z. officinale* effectively attenuates chemotherapy-induced neuropathic pain [31], as cold and mechanical allodynia significantly decreased after the oral treatment of *Z. officinale* in mice. These data let us speculate that ginger and its sub-component could be used to treat different types of pain. Moreover, as it has been reported to not induce any lethal effects [18], if the understanding of the effect and the mechanism of action increases, it could be considered a good option to treat pain. 

Thus, in this review, the effect of *Z. officinale*, [6]-gingerol, and [6]-shogaol has been summarized and analyzed along with the underlying mechanisms of action. This review study includes a total of 16 studies.

## 2. Results

**Figure 1:**
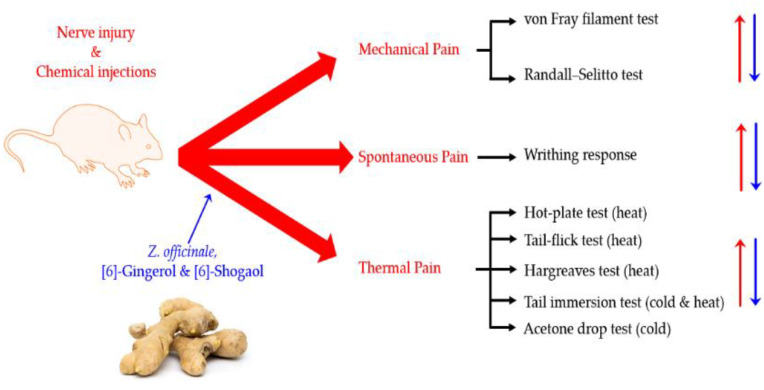
Analgesic effects of *Z. officinale*, [6]-gingerol and [6]-shogaol in mechanical, spontaneous, and thermal pain induced by nerve injury or chemical injection, and a summarization of behavior tests used in the experiment. The pain is induced by a nerve or chemical injection (Red) and alleviated by *Z. officinale* and its sub-components (Blue). Abbreviations: *Z. officinale* (*Zingiber officinale* Roscoe).

This review includes a total of 16 studies (Table 1 and Table 2). Nine studies analyzed the analgesic effect of *Z. officinale* [19,31,32,33,34,35,36,37,38], and four [39,40,41,42] and three [43,44,45] studies focused on [6]-gingerol and [6]-shogaol, respectively. To analyze their effects on different types of pain, studies have been subdivided into three types of pain; mechanical, spontaneous and thermal pain (Figure 1). The mechanical pain section contains seven studies, and the spontaneous and thermal pain section contains four and ten studies, respectively.

### 2.1. Mechanical Pain

Various sensory receptors are present on the skin, such as mechanoreceptors, thermoreceptors and nociceptors [46]. Among them, nociceptors transmit pain signals related to mechanical, thermal, or chemical [47]. Nociceptors include both myelinated and unmyelinated neurons such as Aβ-, Aδ- and C-fiber nociceptors, respectively. Among them, Aβ and Aδ nociceptor neurons are known to mediate mechanical sensation and pain [48]. Mechanical pain could be associated with nerve damage [49] and changed in the activities of various sodium channels (i.e., voltage-gated sodium channel (Na_v_) 1.7 and Na_v_1.8) [50]. In addition, the depression of gamma-Aminobutyric acid (GABA)ergic interneurons increases in the expression of transient receptor potential vanilloid 1 (TRPV1) [51], and the decrease in the potassium channel subfamily K member 1 (TREK-1) channel [52] has also been reported to be the cause of mechanical pain.

To assess the effect of *Z. officinale* and its sub-components on mechanical pain, studies used different types of nerve injury methods, such as chronic constraint injury (CCI) [40], spinal nerve ligation (SNL) [42], spared nerve injury (SNI) [38], and intermittent cold stress (ICS) [35], or chemicals such as acetic acid [41], streptozotocin (STZ) [43,44] and oxaliplatin [31] to induce pain in rodents. Mechanical pain has been evaluated by either von Frey filaments tests [31,38,40,41,42,44,45] or the Randall–Selitto test [35,43].

Nerve-injury-induced animal models of pain have been used by both Gauthier et al. [40], Mata-Bermudez et al. [42] and Borgonetti et al. [38]; however, the method was different, as Gauthier et al. used CCI, whereas Mata-Bermudez et al. and Borgonetti et al. used SNL and SNI animal models of pain, respectively. CCI consists of four loose ligations around the sciatic nerve damaging most of the myelinated neurons but leaving intact the unmyelinated C-fibers. The CCI-induced pain rodents demonstrate spontaneous, thermal, and mechanical pain, which appears from three days to two months after the injury [53]. SNL is the tight ligation of L5-6 spinal nerves. In this model, the degenerative fibers of the damaged roots come into contact with the distal portion of the undamaged roots [54]. In SNL models, L4 dorsal root ganglia (DRG) is unaffected, whereas L5-6 DRG is affected [55]. Pain occurs quickly after nerve ligation and lasts at least four months [56]. In SNI-induced pain, only the tibial and common peroneal nerves are axotomized, leaving the sural nerve intact. The undamaged fibers are in contact with the proximal part of the injured nerves [57]. SNI models differ from other surgery models in that they can examine distinct regions of the hind paw that are innervated by damaged or undamaged neurons. In addition, this model has been demonstrated to closely mimic many features of clinical neuropathic pain. SNI showed pain 24 h after surgery and reached its peak about two weeks later [58].

Nerve injury models such as CCI, SNL, SNI and partial sciatic nerve ligation (PSNL) models all measure the cutaneous sensory threshold of ipsilateral hind limb and these pains are evaluated mainly by thermal and mechanical stimuli [56,59]. 

Gauthier et al. [40] has reported that [6]-gingerol could effectively attenuate mechanical pain induced by CCI. The pain lasted from 1 to 10 days after the surgery, and intrathecally administered 10 µg of [6]-gingerol demonstrated an analgesic effect, which lasted till 4 h after the injection. In the study of Mata-Bermudez et al. [42], the same dose of [6]-gingerol also attenuated SNL-induced mechanical pain. The anti-analgesic effects of [6]-gingerol initiated 60 min after the administration, which gradually decreased after four hours. They further reported that various serotonin (5-HT) receptors, such as 5-HT_1A_, _1B_, _1D_ and _5A_, but not opioid receptors, are involved in the analgesic effect of [6]-shogaol. In addition, in their study, intrathecal pre-treatment of nonselective nitric oxide (NO) synthase inhibitor, inhibitor of guanylate cyclase, and ATP-sensitive K^+^ channels channel blocker also inhibited the [6]-gingerol-induced anti-allodynic effect.

Borgonetti et al. [38] used SNI-induced animal models of pain to confirm the analgesic effect of single and multiple administration of *Z. officinale*. First, the acute oral administration of *Z. officinale* significantly increased the threshold to mechanical stimuli, which was reduced after surgery. In their second experiment, the repeated oral administration of *Z.* *officinale* for 7 days starting from 3 days after surgery significantly decreased the pain induced by mechanical stimuli. Among the three doses used in the study (100, 200 and 400 mg/kg), the anti-allodynia effect of 200 mg/kg was greater, which was similar to 30 mg/kg of pregabalin. The increase in histone deacetylase 1 (HDAC1) in BV2 cell and spinal cord after nerve injury were not shown in single and repeated *Z. officinale* treated rodents. Moreover, acute *Z. officinale* application decreased both phosphorylated extracellular signal-regulated kinase 1/2 (pERK1/2) activation in BV2 cell and spinal cord, respectively; however, repeated *Z. officinale* treatments decreased pERK2 activation in the spinal cord. Montserrat-de la Paz et al. [35] did not use a surgical model, but exposed rodents to intermittent cold places (ICS) to assess the effect of *Z. officinale* against mechanical pain. *Z. officinale* (0.5 and 1%) was given in combination with the standard diet that initiated eight weeks before inducement of hyperalgesia, and the result shows that it dose-dependently alleviated mechanical pain. In their study, paracetamol was also treated in combination with *Z. officinale* and the co-administration-treated group mice showed less pain than individually administered littermates.

Contrasting to the above-mentioned studies, Lee et al. [31] demonstrated the effect of *Z. officinale* in chemotherapy-induced mechanical pain. As a chemotherapeutic agent, they used oxaliplatin (single, intraperitoneal injection, i.p.; 6 mg/kg), which is a widely used anti-cancer agent to treat colorectal and breast cancer. Mechanical pain induced by oxaliplatin lasted from three to five days after the injection. *Z. officinale* was orally administrated for three days after oxaliplatin injection and *Z. officinale* significantly attenuated mechanical pain for 1 h. In addition, to confirm the mechanism of the analgesic effect of *Z. officinale*, Lee et al. focused on the role of serotonin receptors present in the spinal cord, as various serotonin receptors are reported to take part in pain pathways. Intrathecal injections of 5-HT_1A_ receptor antagonist before the treatment of *Z.* *officinale* blocked its analgesic effect. Moreover, the spinal expression of the 5-HT_1A_ receptor was significantly decreased after oxaliplatin injection, whereas *Z. officinale* treatment reversed the decreased mRNA expression level of the 5-HT_1A_ receptor. In addition, Kim et al. [45] also reported that [6]-shogaol could significantly attenuates mechanical pain in neuropathic pain induced by oxaliplatin as in the study of Lee et al. [31]. In this experiment, [6]-shogaol was intraperitoneally injected four days after oxaliplatin injection. One hour after the administration of [6]-shogaol, the threshold to mechanical stimuli was significantly increased compared to that of the oxaliplatin group. As the mechanism of action of [6]-shogaol, authors have demonstrated that the effect of [6]-shogaol was blocked by the intrathecal injection of 5-HT_1A, 3_ and GABA_B_ receptor antagonists. Moreover, treatment of [6]-shogaol increased spinal GABA and glutamate decarboxylase 65 (GAD65) protein concentration in the spinal dorsal horn of L4–5 segments. Altogether, these results suggest that *Z. officinale* and its sub-components use spinal serotonergic pathways to induce an analgesic effect.

In two studies conducted by Fajrin et al. [43,44], STZ-induced animal models of diabetic pain were used to assess the pain-decreasing effect. In their first study [43], *Z. officinale* and [6]-shogaol significantly attenuated mechanical pain induced by 110 mg/kg of STZ injection. Moreover, both *Z. officinale* and [6]-shogaol demonstrated less damage in the sciatic nerve’s morphology compared to the STZ group. In their second study [44], both *Z. officinale* and [6]-shogaol significantly decreased mechanical pain induced by STZ injection. They reported that both *Z. officinale* and [6]-shogaol could significantly reduce upregulated spinal TRPV1 and N-methyl D-aspartate receptor subtype 2B (NMDAR2B) mRNA expression after STZ treatment.

Hitomi et al. [41] assessed the effect of [6]-gingerol and [6]-shogaol in 50% acetic acid filter paper-induced oral ulcerative mucositis (OUM) pain rats. In this study, the swab application of 300 and 150 μM of [6]-gingerol and [6]-shogaol, respectively, failed to attenuate the pain. However, when 13.5 mg/mL of ginseng was applied together, the mechanical threshold significantly increased and spontaneous mouth rubbing decreased Additionally, both [6]-shogaol and [6]-gingerol at 100 μM exhibited significant antagonistic effects on the Na_v_1.8 currents and decreased substance P (SP) release induced by KCL and veratridine in CHO cells.

In summary, the above-mentioned studies demonstrate that *Z.* *officinale* and its main physiological active indicators, [6]-gingerol and [6]-shogaol, could significantly attenuate mechanical pain that has been induced by various animal models of pain. 

### 2.2. Spontaneous Pain

Spontaneous pain includes sensations of stabbing, shooting, burning and paroxysmal pain associated with dysesthesia or paresthesia [60]. Paresthesia and dysesthesia, one of the symptoms of neuropathic pain, is spontaneous, and the cause of this sensation seems to be a spontaneous firing of nerve sprouts that changed the innervation area of peripheral nerves, and sensitization of Aβ and C-fibers [60]. However, it is still unclear whether A- or C-fibers, injured or uninjured fibers, are more important for spontaneous pain generation [61]. It has been also reported that ethological activity in nerve-end neuroma, DRG, and the thalamus can be the basis for spontaneous pain [62]. Chronic inflammatory and neuropathic pain is clinically characterized by a type of spontaneous pain [63].

In this section, various types of chemicals, such as acetic acid [19,32,39], formalin [39] and allyl isothiocyanate (AITC) [37] were used to induce spontaneous pain in rodents, and writhing or licking response was measured to assess the spontaneous pain [19,32,37,39].

Y et al. [32], Ojewole [19] and Young et al. [39] all used acetic acid to induce spontaneous pain in mice. Intraperitoneal injection of acetic acid is known to cause inflammation of the abdominal cavity and induce writhing behavior due to visceral stimulus [64]. Y et al. [32] reported that *Z. officinale* could prevent acetic acid-induced spontaneous pain in mice. Spontaneous pain was induced by intraperitoneal injection of 3% acetic acid (i.p.), and increase in the number of abdominal constrictions (writhing) and stretching with a jerk of the hind limb were shown after the injection. *Z. officinale* was intraperitoneally injected 1 h before acetic acid administration, and it significantly prevented acetic acid-induced writhing. The effect of *Z. officinale* was similar to the effect of 150 mg/kg of aspirin, which was used as a positive control. In the work of Ojewole [19], writhes induced with acetic acid were recorded for 20 min after intraperitoneal injection of 3% acetic acid. *Z. officinale* was administrated (i.p.) 20 min preceding the acetic acid injection, and it significantly decreased acetic acid-induced writhes.

Young et al. [39] reported that [6]-gingerol has an analgesic effect in both acetic acid and formalin-induced spontaneous pain in mice. Five minutes after intraperitoneal injection of 1% acetic acid, the number of writhing increased during the following ten min. [6]-gingerol was injected intraperitoneally 30 min prior to acetic acid injection, and it significantly attenuated the writhing response. In their subsequent study, 1% formalin (20 μL) was injected to the dorsal surface of the right hind-paw to induce spontaneous pain, and the amount of time spent licking or biting the hind-paw was recorded for 40 min. The formalin test is divided into early and late phases. The early phase is caused by C-fiber activation due to peripheral stimulation, and the late phase is known to be caused by inflammatory reactions in peripheral tissues and functional changes in spinal dorsal horn [65]. [6]-gingerol and indomethacin were, respectively, administered 30 min before formalin injection. Both [6]-gingerol and indomethacin significantly attenuated the late phase (period between 15 and 40 min post formalin injection), but not the early phase (first 5 min post formalin injection) of 1%-formalin-induced licking time.

In the study of Kravchenko et al. [37], external application of *Z. officinale* as ointments, attenuated the AITC-induced spontaneous pain. AITC (0.5%, 20 μL) was injected in the sub plantar region of mice to induce spontaneous pain, and a total time spent by the animal on licking the affected limb was observed for ten minutes. *Z. officinale* ointment was applicated five to ten minutes before the injection of AITC, and a different concentration of *Z.* *officinale* extracts showed an analgesic effect in the group that applied ointments ten minutes before the AITC injection. Among them, 0.05% ointment observed the highest level of analgesic activity.

Altogether these four studies suggest that *Z. officinale* and [6]-gingerol could be used to attenuate the spontaneous pain induced with acetic acid and formalin injection as the writhing and licking the affected limb decreased as much as the conventionally used drugs, such as aspirin [66], diclofenac [19] and indomethacin [39], which were used as positive controls in the included studies.

### 2.3. Thermal Pain

Thermal pain is a common symptom both to neuropathic pain caused by nerve injury and systemic inflammatory disorders [67,68]. It refers to a change in perception of temperature, which increases sensitivity to noxious heat or cold and it also typically involves recognizing “warm” or “cold” stimuli as painful [66]. C-fiber nociceptors, non-myelinated neurons among nociceptors present in the skin, are known to mediate thermal pain sensitivity [48]. In addition, the behavioral detection response (i.e., a stabbing pain caused by heat and cold) induced by harmful radiant skin heating appears to also be mediated by Aδ nociceptor activation [69]. The reaction of myelinated Aδ-fibers to noxious heat indicates a sense of pain at a threshold of 43 to 45 °C [70], whereas C-fiber nociceptors have a pain sensing threshold value of 41 °C on average [71]. TRPV1, also known as the capsaicin receptor, is known as the major molecular transducer of polymodal nociceptors that detect heat [72]. In humans, the innocuous cold mainly activates myelinated Aδ-fibers, and the noxious cold activates both polymodal C-fibers and Aδ-fibers. Additionally, transient receptor potential melastatin 8 (TRPM8), a non-selective cation channel, is known as the main mechanism of cold sensing in peripheral neurons [73].

In this section, thermal pain was induced by nerve injury (i.e., CCI [40], PSNL [36], ICS [35] and SNI [38]) or chemical (i.e., complete freund’s adjuvant (CFA) [36], STZ [43,44] and oxaliplatin [31,45]) injections, and thermal pain was measured by using hot-plate [19,35,36,44], tail-flick [33,34,43], hargreaves [38,40], immersion [35] and acetone drop tests [31,45].

Three studies observe the effect of *Z.* *officinale* and [6]-gingerol in nerve-injury-induced thermal pain (SNI, CCI, and PSNL). First, Borgonetti et al. [38] demonstrated the analgesic effect of *Z.* *officinale* in SNI-induced thermal pain in mice. Heat pain was evaluated by using hargreaves’ plantar test. SNI-induced thermal pain lasted till 21 days after the nerve injury. *Z.* *officinale* was injected orally at day seven after surgery, and 200 mg/kg of *Z.* *officinale* completely attenuated the heat pain. The analgesic effect of 200 mg/kg *Z.* *officinale* was similar to that of the pregabalin. Second, Gauthier et al. [40] reported the effect of [6]-gingerol in CCI-induced thermal pain. Thermal hyperalgesia was evaluated by hargreaves test, and tests were conducted at 30 min, 2 h and 4 h following intrathecal injections of [6]-gingerol (10 µg) on both paws. The results show that [6]-gingerol could attenuate thermal hyperalgesia from 30 min to 2 h and 4 h after its administration. Finally, Fajrin et al. [36] analyzed the effect of *Z.* *officinale* in PSNL- and CFA-induced neuropathic and inflammatory pain mice, respectively. The PSNL model ligates 1/3–1/2 of the sciatic nerve to induce pain, and it is known to be associated with the development of spontaneous pain, allodynia and hyperalgesia. However, it is difficult to associate PSNL injuries with specific DRG or spinal levels due to a random mixture of injured L4-5 spinal nerves [74]. *Z.* *officinale* was orally injected once a day for seven consecutive days a week after the inducement of heat pain by CFA injection and PSNL. Their results show that *Z. officinale* administration significantly increased the latency time toward thermal stimulus. The 200 mg/kg dose was the most effective in PSNL-induced neuropathy pain, whereas the 400 mg/kg dose was the most effective in CFA-induced inflammatory pain. Montserrat-de la Paz et al. [35] used ICS-induced FMS models to observe the effect of *Z. officinale* on thermal pain. Symptoms of FMS include thermal allodynia or hyperalgesia, and hot plate test or tail immersion test was used for evaluation, respectively. *Z. officinale* (0.5 and 1%) and paracetamol were supplied in combination with the standard diet daily that initiated eight weeks prior the inducement of pain. In the hot plate test, only *Z. officinale* (0.5%) and co-administrated group significantly decreased the thermal hyperalgesia. However, in the tail immersion test, the *Z. officinale* (0.5 and 1%) alone group was effective in both cold and hot pain (allodynia and hyperalgesia).

Chemotherapy treatment is also known to induce thermal pain both in humans and rodents [75,76]. In the study of Lee et al. [31] and Kim et al. [45], cold pain was assessed by using the acetone drop test. Lee et al. [31] injected different doses of *Z.* *officinale* orally in oxaliplatin-induced neuropathic pain, and all doses succeeded in significantly attenuating cold pain when measured 60 min after its administration. Kim et al. [45] also reported that [6]-shogaol could significantly alleviate cold pain in neuropathic pain induced by oxaliplatin. [6]-shogaol was injected intraperitoneally, and analgesic effect was shown 60 min after the administration. Fajrin et al. reported two studies related to thermal pain; on the first study [43], the efficacy of *Z. officinale* and [6]-shogaol were evaluated through a tail-flick test in the STZ-induced heat pain in mice. Oral and intraperitoneal administration of *Z. officinale* and [6]-shogaol, decreased thermal hyperalgesia, respectively. In their subsequent study [44], STZ was also used to induce thermal pain (heat), and the hot-plate test was used to evaluate the analgesic effect of *Z. officinale* and [6]-shogaol. The results show that both *Z. officinale* and [6]-shogaol treated group mice showed significantly longer latency time toward thermal stimulus compared to the diabetic control group.

Ojewole [19] and Sepahvand et al. [33] evaluated the effect of *Z. officinale* in electrical and radiant heat-induced thermal pain using a hot plate test and a tail flick test, respectively. In the study of Ojewole, *Z. officinale* was intraperitoneally administrated 20 min before the hot-plate test, and jumping-out of the beaker was considered a response to heat-induced pain. *Z. officinale* treatment significantly delayed the reaction time induced by electrical heat. Sepahvand et al. [33] also demonstrated the effect of *Z. officinale* through a tail-flick test in radiant heat-induced pain in rats. The tail-flick test was evaluated after intraperitoneal injection of the *Z. officinale* or morphine. *Z. officinale* was injected 15 min before morphine injection to confirm the effect of co-administration in morphine analgesia. *Z. officinale* exerted an analgesic effect in tail-flick test, which peaked at 30 min after injection and lasted till 60 min. The analgesic effect of *Z. officinale* peaked at 30 min after the injection and lasted till 120 min, respectively (120 mg/kg). Morphine alone showed no analgesic effect; however, co-administration of *Z. officinale* (200 mg/kg) and morphine produced an antinociceptive effect that lasted 120 min. As a result, the analgesic effect of *Z.* *officinale* alone or with morphine was greater than the morphine.

Darvishzadeh-Mahani et al. [34] have reported that *Z. officinale* could protect the development of morphine-induced tolerance in radiant heat-induced pain (tail-flick test). The tolerance of analgesic effect was demonstrated by multiple injections of morphine (twice a day for eight days). *Z. officinale* was given through the oral route and co-administered with morphine. Concomitant treatment of morphine and *Z. officinale* significantly prevented the morphine-induced tolerance. Dose of 25 mg/kg of *Z. officinale* shows anti-tolerance effect, whereas 10 mg/kg *Z. officinale* failed to show a significant effect. In addition, co-administration of morphine and *Z. officinale* (100 mg/kg) reversed the morphine-induced L-type calcium channel over-expression in the spinal cord.

Altogether, the results demonstrated in the included studies clearly show that *Z. officinale*, [6]-gingerol and [6]-shogaol can effectively attenuate thermal pain (i.e., cold and heat) induced by nerve injury and chemotherapy treatment.

## 3. Discussion

In this study, the effect of *Z. officinale*, [6]-gingerol and [6]-shogaol on different types of pain have been summarized. A total of 16 studies that focused on *Z.* *officinale* [19,31,32,33,34,35,36,37,38], [6]-gingerol [39,40,41,42] and [6]-shogaol [43,44,45] have been included. To our knowledge, this is the first time that their effect and underlying mechanism of action in pain have been analyzed. *Z. officinale* is widely known for its effect on the digestive system, and it has been mainly used to treat digestive disorders [77,78,79]; however, recent clinical [80,81] and animal [82,83,84] studies suggest that it could also be effective against the pain, but too little is known on their effect and mechanisms of action.

*Z. officinale*, ginger, which has long been widely used to treat various diseases, is one of the most popular herbal dietary supplements in the world [85]. It is also known to cause no severe side effects, and the U.S. Food and Drug Administration (FDA) classified ginger as “generally recognized as safe” [86]. The components of *Z. officinale* include volatile oils, fixed fatty oils and pungent compounds but depends on the characteristics of the cultivated region, agroclimatic conditions [87]. As the pungent compounds, [6]-gingerol and [6]-shogaol, are the two main compounds [88]. When gingerol, which is unstable in heat, is deformed at a high temperature, it becomes shogaol, and [6]-shogaol is the most common dehydrated product [89]. Although the content of [6]-gingerol and [6]-shogaol in *Z.* *officinale* appears to be affected by drying and extraction temperatures [89], it is reported that about 11% and 0.08% are contained in *Z.* *officinale*, respectively [90]. Both shogaols and gingerols are known to easily pass the blood–brain barrier (BBB) [91].

In this study, the analgesic effect of *Z.* *officinale*, [6]-gingerol and [6]-shogaol have been analyzed on mechanical, spontaneous, and thermal allodynia or hyperalgesia (Table 1 and Table 2), and different animal models of pain have been used. Among the 16 studies included, five used different types of nerve injury pain models, whereas 11 used diverse chemicals to induce pain in rodents. On mechanical allodynia, five studies focused on the effect of *Z. officinale* and four on [6]-shogaol and three on [6]-gingerol. On spontaneous pain, three observed the pain-decreasing effect of *Z. officinale* and one of [6]-gingerol. Finally, on thermal pain, nine reported the action of *Z. officinale* and one and three of [6]-gingerol and [6]-shogaol, respectively.

**Figure 2:**
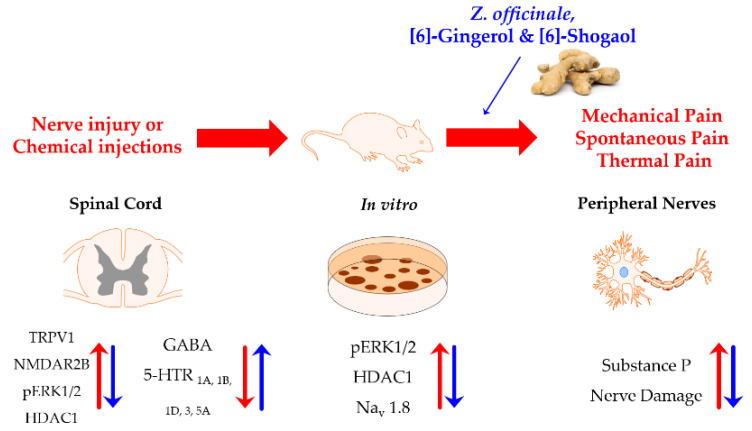
The pathogenesis mechanism of pain induced by nerve injury or chemical injection and the mechanism of action of the analgesic effect of *Z. officinale*, [6]-gingerol and [6]-shogaol. Pain is caused by nerve injury or chemical injection (Red), and pain is attenuated when *Z. officinale* and its sub-components are administered (Blue). Mechanism was identified on the spinal cord, peripheral nerves and cultured cell (in vitro). Abbreviations:5-HTR (serotonin receptor), GABA (gamma-aminobutyric acid), HDAC1 (histone deacetylase 1), Na_v_1.8 (voltage-gated sodium channel 1.8), NMDAR2B (N-methyl-D-aspartate receptor subunit 2B), pERK (phosphorylated extracellular signal-regulated kinase), TRPV1 (transient receptor potential vanilloid 1), and *Z. officinale* (*Zingiber officinale* Roscoe).

In the included studies, only seven papers have demonstrated the underlying mechanism of action of the analgesic effects of *Z.* *officinale*, [6]-gingerol and [6]-shogaol [31,38,41,42,43,44,45]. Five studies have focused on the spinal cord, one on the DRG neurons and one has used cultured cell. Three studies [31,42,45] focused on the role of spinal serotonergic receptors [31,42,45], and spinal TRPV1, spinal NMDA receptor (NMDAR) [44], spinal pERK1/2, histone deacetylase (HDAC1) [38], spinal pERK1/2, histone deacetylase (HDAC1), sciatic nerve’s morphology [43], and Na_v_1.8 and SP [41] have been observed by one study (Figure 2).

To assess the involvement of the serotonergic system, Lee et al. [31], Kim et al. [45] and Mata-Bermudez et al. [42] observed the role of serotonergic receptors in the spinal cord. On the oxaliplatin-induced animal model of pain, both Lee et al. and Kim et al. have reported that intrathecal pre-treatment of 5-HT_1A_ and 5-HT_3_ receptor antagonists could block the analgesic effect of *Z. officinale* and [6]-shogaol. Although the animal model of pain was different (oxaliplatin vs. SNL), Mata-Bermudez et al. have also focused on spinal 5-HT_1A_, _B_, _D_, and _5A_ receptors and demonstrated that the analgesic effect of intrathecal injection of [6]-gingerol is mediated by these receptors. In addition, in the study of Kim et al. [45], [6]-shogaol was shown to decrease both the mechanical and cold pain through spinal 5-HT_1A_ and 5-HT_3_ receptors present in the spinal GABA neurons, which are inhibitory interneurons [45]. Altogether, these results suggest that both *Z. officinale* and [6]-shogaol act on spinal 5-HT_1A_ and 5-HT_3_ receptors and [6]-gingerol on spinal 5-HT_1A_, _B_, _D_, and _5A_ receptors. Seven families of serotonin recipients are divided into 15 subtypes [92], and are found in both central and peripheral nervous systems [93]. Among them, 5-HT_3_ receptors are ligand-gated ion channels (LGICs), whereas other receptors are G-protein-coupled receptors (GPCRs) [92]. 5-HT_1_, _3_ and _5_ receptors are known to be present in the superficial laminae of the dorsal horn of the spinal cord and are reported to induce an analgesic effect upon activation [94,95,96]. Although the included studies have demonstrated that *Z. officinale* and its sub-components could induce analgesic effect through serotonergic receptors present in the spinal cord, much remains to be clarified, as whether they directly activated these receptors or indirectly activated them by increasing the synthesis of descending serotonin from the rostro ventromedial medulla (RVM) of the brain has not been understood yet. Thus, further studies are needed to clearly understand the role of the serotonergic system in the analgesic effect of *Z. officinale* and its sub-components.

In the study conducted by Fajrin et al. [44], the role of spinal TRPV1 has been observed. [6]-Gingerol and [6]-shogaol are known as capsaicin structural analogs [97] and have a high binding affinity for TRPV1 [98]. By using a diabetic induced animal model of pain, Fajrin et al. has reported that *Z. officinale* and [6]-shogaol modulate the expression of spinal TRPV1 to induce analgesia. They reported that both *Z. officinale* and [6]-shogaol decrease the expression of TRPV1 in the spinal cord. Compared to the relatively well understood role of the TRPV1 present on the peripheral nervous system, the role of spinal TRPV1 has not been clearly understood yet [99,100]. In the spinal cord, TRPV1 is known to exist in the superficial laminae I and II, which are pain sensory pathways [101]. Kanai et al. [102] confirmed a gradual increase in TRPV1 expression in superficial dorsal horns of spinal cord in the CCI rats model and reported that intrathecal administration of TRPV1 antagonist could induce analgesia. In addition, mechanical and heat hypersensitivity induced by spinal cord injury were reversed by intrathecal injection of antisense oligonucleotide, which knockdown spinal TRPV1 [103]. In clinical trials, the TRPV1 antagonist has been reported to significantly increase the threshold for capsaicin-induced heat and pressure pain in healthy volunteers [104]. TRPV1 has also been reported to be related to the activity of spinal astrocytes [105] and microglia [106] augmenting the ascending neuronal pain signals transmitted to the brain. Furthermore, TRPV1 can interact with NMDAR2B to contribute to pain development [107], as a study has reported that spinal TRPV1 expression was increased in carrageenan-inducted pain condition, and expression of TRPV1 and phosphorylated NMDAR2B decreased when capsazepine, the TRPV1 antagonist, was intrathecally administered [108]. Furthermore, *Zingiber zerumbet*, which is a different species of the Zingiberaceae family [109], has also shown an antinociception effect similar to capsazepine [110]. They further revealed that the antinociception effect of *Zingiber zerumbet* is mediated through the NO and adenosine triphosphate (ATP)-sensitive K^+^ channel pathway. The opening of the ATP-sensitive K^+^ channel, which releases K^+^, leads to a decrease in membrane excitability through membrane repolarization or hyperpolarization [111]. Similarly, Mata-Bermudez et al. [42] have demonstrated that [6]-gingerol affected the NO–cyclic guanosine monophosphate–ATP-sensitive K^+^ channel pathway to induce analgesia. In addition to the above-mentioned mechanisms, calcitonin gene-related peptide (CGRP) has been reported to be modulated by *Z. officinale,* as an in vitro study has suggested that *Z. officinale* could attenuate the trigeminal pain by modulating CGRP [112]. CGRP is known as the main inflammatory mediator in neurogenic inflammation of migraine. Peripheral release of CGRP is known to be involved in the development and maintenance of central sensitization and allodynia, and receptor antagonist of CGRP is targeted as a treatment for migraine and chronic pain [113]. TRPV1 expressed in trigeminal nociceptors has also been reported to cause neurogenic inflammation by releasing CGRP [114].

In conclusion, based on the results obtained from 16 studies, our review suggests that *Z. officinale* and its sub-components (i.e., [6]-gingerol and [6]-shogaol), which have long been used as herbal medicines, can be used to treat mechanical, spontaneous, and thermal (cold and heat) pain. However, more studies that focus on the mechanism of action are still needed, as the understanding of the underlying mechanism of action is still poor, especially on the role of the serotonin system and TRPV1. Furthermore, future studies should focus not only on the spinal cord, but also on the brain and the peripheral nervous system to enlarge the understanding on the effect of *Z. officinale.*

## 4. Materials and Methods

**Figure 3:**
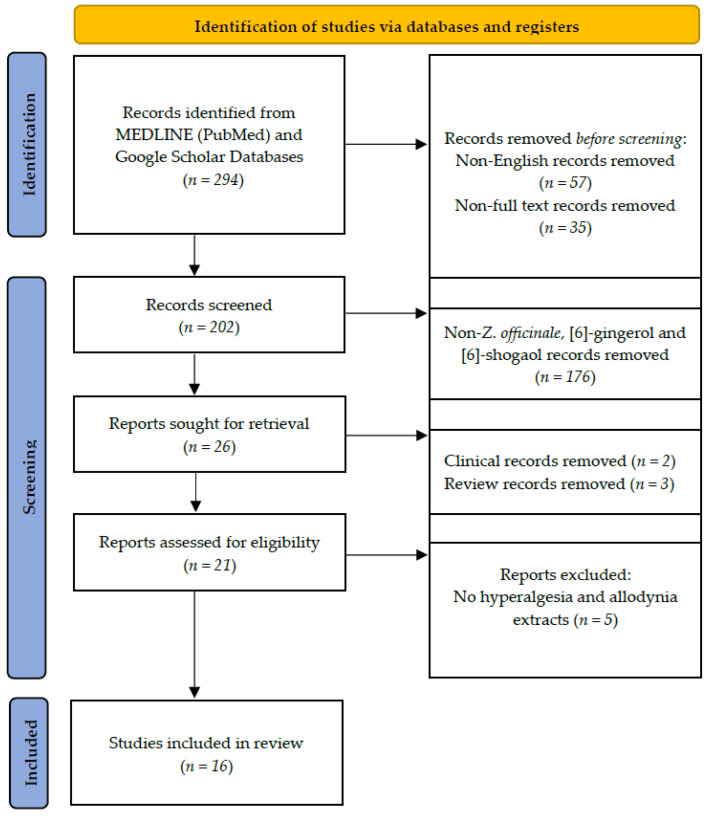
Flow chart of the article-inclusive protocol. Identification through searches of MEDLINE (PubMed) and Google Scholar yielded 294 articles, which were screened by abstract and full-text examinations. Finally, a total of 16 articles analyzing the effect of *Z. officinale*, [6]-gingerol and [6]-shogaol in hyperalgesia and allodynia in rodents were included in our review.

A search was conducted on all studies on the effect of *Z. officinale* and its sub-components of pain in the National Library of Medicine (MEDLINE) using PubMed, and Google Scholar (Figure 3). Extensive searches were undertaken for articles written in English, as non-English studies were excluded. Studies electronically published until the end of June 2022 were included. The literature search was performed using the following keywords: “*Zingiber officinale* roscoe (*Z. officinale*)”, “[6]-Shogaol”, “[6]-Gingerol”, “Allodynia” and “Hyperalgesia” “Pain”. After the initial search, duplicates, bibliographies, study protocols, clinical trials, and non-English studies were excluded. Sixteen animal studies were included in this study.

## Figures and Tables

**Table 1:** Summary on the effect of *Z. officinale* on pain.

Authors	Strain	Pain	*Z. officinale* Roscoe	Findings		
Rats						
Sepahvand et al., 2010 [33]	Wistar Rat	Tail-Flick Test	200, 400 and 600 mg/kg (i.p. 80% Ethanol Extract)	Control:	Heat Pain	-
				*Z. officinale*:		↓
				*Z. officinale* + Morphine (2.5 mg/kg):		↓
				Mechanism of Actions:	-	
Darvishzadeh-Mahani et al., 2012 [34]	Wistar Rat	Tail-Flick Test	50 and 100 mg/kg (p.o. 96% Ethanol Extract)	Control:	Heat Pain	↑
				*Z. officinale*:		↓
				Mechanism of Actions:	-	
Mice						
Y et al., 2002 [32]	Swiss Mice	Acetic Acid 3% (i.p.)	50 and 100 mg/kg (i.p., 100% Ethanol Extract)	Control:	Spontaneous Pain	↑
				*Z. officinale*:		↓
				Aspirin (150 mg/kg, i.p.):		↓
				Mechanism of Actions:	-	
Ojewole 2006 [19]	Balb C Mice	Acetic Acid 3% (i.p.) and Hot Plate Test	100, 200, 400 and 800 mg/kg (i.p. 96% Ethanol Extract)	Control:	Spontaneous and Heat Pain	↑
				*Z. officinale*:		↓
				Morphine (10 mg/kg, i.p.): Diclofenac (100 mg/kg, i.p.):		↓
				Mechanism of Actions:	-	
Montserrat-de la Paz et al., 2018 [35]	C57BL/6J Mice	ICS-induced FMS models	0.5 and 1% (p.o. Mixed with Standard Diet)	Control:	Cold, Heat and Mechanical Pain	↑
				*Z. officinale:*		↓
				*Z. officinale* + Paracetamol:		↓
				Mechanism of Actions:	-	
Fajrin et al., 2019 [36]	Mice	CFA 40 μL (Intraplantar Injection) and PSNL	100, 200, 400 and 600 mg/kg (p.o., Destilator with Aquadest)	Control:	Heat Pain	↑
				*Z. officinale*:		↓
				Mechanism of Actions:	-	
Kravchenko et al., 2019 [37]	White Mice	AITC 0.5% (Subplantar Injection)	0.0125, 0.025, 0.05, 0.1, 0.5, 1 and 5% of Extract Ointment	Control:	Spontaneous Pain	↑
				*Z. officinale*:		↓
				Benzocaine (Ointment):		↓
				Mechanism of Actions:	-	
Fajrin et al., 2019 [43]	Balb/c Mice	STZ 110 mg/kg (i.p.)	100, 200 and 400 mg/kg (p.o., 96% Ethanol Extract)	Control:	Heat and Mechanical Pain	↑
				*Z. officinale*:		↓
				Gabapetin (100 mg/kg, p.o.):		↓
				Mechanism of Actions:	Prevention of sciatic nerve damage	
Fajrin et al., 2020 [44]	Balb/c Mice	STZ 110 mg/kg (i.p.)	100, 200 and 400 mg/kg (p.o., 96% Ethanol Extract)	Control:	Heat and Mechanical Pain	↑
				*Z. officinale*:		↓
				Gabapetin (100 mg/kg, p.o.):		↓
				Mechanism of Actions:	↓ TRPV1 and NMDAR2B mRNA expression (spinal cord)	
Borgonetti et al., 2020 [38]	CD1 Mice	SNI	200 and 400 mg/kg (p.o., Supercritical CO_2_ extraction)	Control:	Mechanical and Heat Pain	↑
				*Z. officinale*:		↓
				Pregabalin (30 mg/kg, p.o.):		↓
				Mechanism of Actions:	↓ pERK1/2 activation (in BV2 cells and spinal cord) ↓ HDAC1 expression (in BV2 cells and spinal cord)	
Lee et al., 2021 [31]	C57BL/6 Mice	Oxaliplatin 6 mg/kg (i.p.)	100, 300 and 500 mg/kg (p.o., 100% Water Extract)	Control:	Cold and Mechanical Pain	↑
				*Z. officinale*:		↓
				Mechanism of Actions:	Analgesic Effect Blocked by Mixed 5-HT_1_ and 5-HT_2_ receptor, 5-HT_1A_ and 5-HT_3_ antagonists’ injections (i.t.) ↑ mRNA expression level of 5-HT_1A_ receptor	

Abbreviations: 5-HT (serotonin), AITC (allyl isothiocyanate), CFA (completed Freud’s Adjuvant), FMS (fibromyalgia syndrome), GR (ginger rhizome), HDAC (histone deacetylase), ICS (intermittent cold stress), i.p. (intraperitoneal), i.t. (intrathecal), NMDAR2B (N-methyl-D-aspartate receptor subunit 2B), mRNA (messenger RNA), pERK (phosphorylated extracellular signal-regulated kinase), p.o. (per os), PSNL (partial sciatic nerve ligation), SNI (spared nerve injury), STZ (streptozotocin), TRPV1 (transient receptor potential vanilloid 1), and *Z. officinale* (*Zingiber officinale* Roscoe).

**Table 2:** Summary on the effect of [6]-gingerol and [6]-shogaol on pain.

Authors	Strain	Pain	Treatments	Findings		
Rats						
Gauthier et al., 2012 [40]	SD Rat	CCI	[6]-Gingerol 10 μg (i.t.)	Control:	Heat and Mechanical Pain	↑
				[6]-Gingerol:		↓
				Cyclodextrin Formulation (20 μL, i.t.):		↑
				Mechanism of Action:	-	
Hitomi et al., 2017 [41]	Wistar Rat	OUM	[6]-Shogaol 150 μM [6]-Gingerol 300 μM (Swab Application)	Control:	Mechanical Pain	↑
				[6]-Shogaol + [6]-Gingerol:		-
				Mechanism of Action:	↓ Evoked currents on Na_v_1.8. (CHO cell) ↓ SP release (CHO cells)	
Mata-Bermudez et al., 2018 [42]	Wistar Rat	SNL	[6]-Gingerol 1, 3, 6 and 10 μg/rat (i.t.)	Control:	Mechanical Pain	↑
				[6]-Gingerol:		↓
				Gabapentin (100 μg/rat, i.t.):		↓
				Mechanism of Action:	Effect not blocked by nonselective opioid receptor antagonist (naloxone, i.t.) Effect blocked by nonselective 5-HT, 5-HT_1A_, _1B_, _1D_, _5A_ receptor antagonists (methiothepin, WAY-100635, SB-224289, BRL-15572, SB-659551, i.t.) Effect blocked by nonselective NO synthase inhibitor, inhibitor of guanylate cyclase, channel blocker of ATP-sensitive K^+^ channels (L-NAME, ODQ, glibenclamide, i.t.)	
Mice						
Young et al., 2005 [39]	ICR Mice	Acetic Acid 1% (i.p.) and 10% Formalin (s.c.)	[6]-Gingerol 25 and 50 mg/kg (i.p.)	Control:	Spontaneous Pain	↑
				[6]-Gingerol:		↓
				Indomethacin (10 mg/kg, i.p.):		↓
				Mechanism of Action:	-	
Fajrin et al., 2019 [43]	Balb/c Mice	STZ 110 mg/kg (i.p.)	[6]-Shogaol 5, 10 and 15 mg/kg (p.o.)	Control:	Heat and Mechanical Pain	↑
				[6]-Shogaol:		↓
				Gabapentin (100 mg/kg, p.o.):		↓
				Mechanism of Action:	Prevention of sciatic nerve damage	
Fajrin et al., 2020 [44]	Balb/c Mice	STZ 110 mg/kg (i.p.)	[6]-Shogaol 5, 10 and 15 mg/kg (p.o.)	Control	Heat and Mechanical Pain	↑
				[6]-shogaol		↓
				Gabapentin (100 mg/kg, p.o.)		↓
				Mechanism of Action	↓ TRPV1 and NMDAR2B mRNA expression (spinal cord)	
Kim et al., 2022 [45]	C57BL/6 Mice	Oxaliplatin 6 mg/kg (i.p.)	[6]-Shogaol 10 mg/kg (i.p.)	Control:	Cold and Mechanical pain:	↑
				[6]-shogaol:		↓
				Mechanism of Action:	Effect blocked by 5-HT_1A_, _3_ receptor antagonists (NAN-190, MDL-72222, i.t.) Effect blocked by GABA_B_ receptor antagonist (CGP 55845, i.t.) ↑ GABA and GAD65 concentration (spinal cord)	

Abbreviations: 5-HT (serotonin), ATP (adenosine triphosphate), GABA (gamma-aminobutyric acid), GAD65 (glutamate decarboxylase 65), i.p. (intraperitoneal), i.t. (intrathecal), L-NAME (Nω-nitro-L-arginine methyl ester), NMDAR2B (N-methyl-D-aspartate receptor subunit 2B), NO (nitric oxide), ODQ (1H-[1,2,4]oxadiazolo [4,3-a]quinoxalin-1-one), OUM (oral ulcerative mucositis), p.o (per os), CCI (chronic constriction injury), SNL (spinal nerve ligation), SP (substance P), STZ (streptozotocin), TP (test pulse), and TRPV1 (transient receptor potential vanilloid 1).

## Data Availability

Not applicable.
